# Evaluation of the Inhibitory Effect of *Moringa oleifera* Leaves Methanolic Extract against *In Vitro* Growth of Several *Babesia* Species and *Theileria equi* and the *In Vivo* Growth of *Babesia microti*

**DOI:** 10.1155/2023/4285042

**Published:** 2023-10-31

**Authors:** Mohamed Abdo Rizk, Shimaa Abd El-Salam El-Sayed, Mohamed Z. Sayed-Ahmed, Yosif Almoshari, Saad S. Alqahtani, Sarfaraz Ahmad, Nawazish Alam, Basma H. Marghani, Abdelbaset E. Abdelbaset, Ikuo Igarashi

**Affiliations:** ^1^Department of Internal Medicine and Infectious Diseases, Faculty of Veterinary Medicine, Mansoura University, Mansoura 35516, Egypt; ^2^National Research Center for Protozoan Diseases, Obihiro University of Agriculture and Veterinary Medicine, Inada-Cho, Obihiro, Hokkaido 080-8555, Japan; ^3^Department of Biochemistry and Chemistry of Nutrition, Faculty of Veterinary Medicine, Mansoura University, Mansoura 35516, Egypt; ^4^Pharmacy Practice Research Unit, Department of Clinical Pharmacy, College of Pharmacy, Jazan University, Jazan 45142, Saudi Arabia; ^5^Department of Pharmaceutics, College of Pharmacy, Jazan University, Jazan 45142, Saudi Arabia; ^6^Department of Clinical Pharmacy, College of Pharmacy, King Khalid University, Abha, Saudi Arabia; ^7^Department of Physiology, Faculty of Veterinary Medicine, Mansoura University, Mansoura 35516, Egypt; ^8^Department of Biochemistry, Physiology and Pharmacology, Faculty of Veterinary Medicine, King Salman International University, South of Sinai 46612, Egypt; ^9^Clinical Laboratory Diagnosis, Department of Animal Medicine, Faculty of Veterinary Medicine, Assiut University, Assiut 71515, Egypt

## Abstract

The current study evaluated the inhibitory effect of *Moringa oleifera* leaves methanolic extract (MOL) against the *in vitro* growth of *Babesia bovis* (*B. bovis*), *B. caballi*, *B. bigemina*, and *Theileria equi* (*T. equi*), as well as *in vivo* growth of *B. microti* in mice. Active principles of MOL extract were determined using liquid chromatography mass spectrometry (LC-MS). MOL's anti-piroplasm efficacy was assessed both *in vitro* and *in vivo* using the SYBR Green I fluorescence assay. Every 96 hours, the hematological parameters, including red blood cell count (RBCs; 10^4^/UL), hemoglobin content (HGB; g/dl), and hematocrit percent (HCT; %), in the treated mice were monitored using a Celltac MEK6450 automated hematological analyzer. LC-MS of MOL revealed that the most abundant polyphenolic catechism found in the MOL extract was isoquercetin and rutin. MOL inhibited *B. bovis, B. caballi, B. bigemina*, and *T. equi in vitro* growth in a dose-dependent way, with IC_50_ values of 45.29 ± 6.14, 19.16 ± 0.45, 137.49 ± 16.07, and 9.29 ± 0.014 *μ*g/ml, respectively. MOL's *in vitro* antibabesial activity was enhanced when administrated simultaneously with either diminazene aceturate (DA) or MMV665875 compound from malaria box. In mice infected by *B. microti*, a combination of MOL and a low dose of DA (12.5 mg·kg^−1^) resulted in a significant (*P* < 0.05) reduction in *B. microti* growth. These findings suggest that MOL is an effective herbal anti-piroplasm therapy, especially when combined with a low dosage of either DA or MMV665875.

## 1. Introduction

In the worldwide livestock sector and animal trade, *Babesia* and *Theileria*, which are blood parasites transmitted by ticks and infect erythrocytes, result in considerable economic losses [1]. The disease's symptoms include sickness, lethargy, hemoglobinuria, jaundice, and mortality [2]. The most prevalent parasitic infestations in cattle are *Babesia bovis* (*B. bovis*) and *B. bigemina* [2]. The main causative agents of the disease in horses are *B. caballi* and *Theileria equi* (*T. equi*) [3]. Unfortunately, no laboratory animals are accessible for cattle or equine *Babesia* infections. However, a rodent *Babesia* model infected with *B. microti* is used for evaluation new drugs [4].

Currently used anti-piroplasm drugs; imidocarb dipropionate and diminazene aceturate (DA) did not exhibit complete clearance of the infection from the infected animals [1]. As a result, the search for alternative anti-piroplasm medicines becomes critical. In this regard, the anti-piroplasm efficacy of *Moringa oleifera* leaves (MOL) methanolic extract is evaluated in the present study. *Moringa oleifera* Lam is the most extensively distributed *Moringaceae* family plant, with a wide range of medicinal and dietary benefits all over the world [5]. Indeed, MOL is rich in natural cancer-preventive compounds and is considered a good source of macro- and micronutrients [4]. MOL also has antibacterial [6, 7], antimalarial [7], and anti-trypanosome [8] properties. However, there has been no research work on the efficacy of MOL extracts as an anti-piroplasm therapy. As a result, we investigated MOL's potency as an anti-piroplasm candidate against the *in vitro* growth of bovine cattle and equine piroplasm parasites, in addition to *B. microti* infection in mice.

## 2. Materials and Methods

### 2.1. Ethical Approval

The Animal Care and Use Committee at Obihiro University of Agriculture and Veterinary Medicine gave its approval to each study's experimental protocols (Approval No. 27–65). The Fundamental Guidelines for the Proper Conduct of Animal Experiments and Related Activities at Academic Research Institutions, published by Japan's Ministry of Education's Culture, Sports, Science, and Technology, were followed in all the experiments. The pathogen experiment's IDs were as follows: (bovine *Babesia*: 201708-4; equine piroplasma parasites: 201910-2; *Babesia microti*: 201709‐05).

### 2.2. Blood Parasites' Growth Inhibition Assays and Drug Combination Tests

MOL powder (bought from https://iherb.com/) was dissolved in 50 mL of 99.8% methanol (Wako Pure Chemical Industries, Ltd., Osaka, Japan) and incubated for 3 days at 30°C. The resulting product was then filtered using Whatman filter paper No. 1 using a rotary evaporator (BUICHI®RotavaporR-200/205, Flawil, Switzerland) and a freeze-drying vacuum system (Labconco, Kansas City, MO, USA) [9–11]. Then, 100 mg (crude extract) was dissolved in 1 mL of DMSO. MOL methanol extracts toxicity to cattle and horse RBCs was assessed at a dosage of 25 mg/ml, as previously published [6]. Active principles of MOL extract were evaluated using liquid chromatography mass spectrometry (LC-MS) [7].

MOL was evaluated against the growth of *B. bovis* (Texas strain), *B. bigemina* (Argentina strain), *B. caballi* [10], and *T. equi* (United States Department of Agriculture) [10] parasites using a fluorescence spectrophotometer [3, 12]. MOL concentrations of 0.0005, 0.001, 0.005, 0.01, 0.025, 0.50, 1, 5, 10, and 25 mg/ml were utilized. For the *in vitro* investigation, the commonly used antibabesial DA drug was used as a control [12]. As previously described in our study [13], the viability assay was utilized to follow up the *in vitro* regrowth of all screened parasites after MOL treatment was stopped. The delayed-death effect of IC_99_ MOL on *B. bigemina in vitro* growth was also assessed [9].

The inhibitory effect of combination therapy consisting of MOL/DA was investigated against the *in vitro* growth of equine piroplasm and bovine *Babesia* parasites. Furthermore, MOL and potent MMV compounds (MMV665941, MMV396693, MMV006787, MMV665810, MMV007092, MMV085203, MMV666093, and MMV665875) from malaria box [2, 14] were assessed against the *in vitro* growth of *B. bovis* (*Babesia* spp. exhibited the highest IC_50_ among screened bovine *Babesia* parasites). Each experiment was performed in triplicates.

### 2.3. MOL *In Vivo* Inhibitory Assay and *B. microti* PCR Detection in Mice

A fluorescence assay was used to evaluate the *in vivo* inhibitory efficacy of a MOL in mice infected with *B. microti* (Munich strain) [15]. Twenty-five female BALB/c mice (CLEA Japan, Tokyo, Japan) were used and divided equally into five groups. Nontoxic doses from MOL either as monotherapy or combined therapy with DA were selected depending on previous study evaluated the antimalarial Activities of MOL extract against *Plasmodium berghei* ANKA infection in mice [8]. 10 *µ*L of venous blood was collected from the tail of each mouse every four days and used for determination the selected hematological variables using a Celltac MEK-6450 automatic hematological analyzer (Nihon Kohden Corporation, Tokyo, Japan). The potential of the MOL/DA combination to clear the infection by *B. microti* from mice's bodies was tested on day 44 post infection using nested PCR assay targeting the *B. microti* small isoform rRNA (ss-rRNA) gene [4, 9]. Each experiment was repeated two times.

### 2.4. Statistical Analysis

A one-way ANOVA test was performed using GraphPad Prism (version 5.0 for Windows; GraphPad Software, Inc., San Diego, CA, USA) to detect the significant differences between the analyzed groups. Statistics were significant at *P* values <0.05.

## 3. Results

### 3.1. MOL Methanolic Extract Inhibits the *In Vitro* Growth of Babesia and Theileria

**Figure 1:**
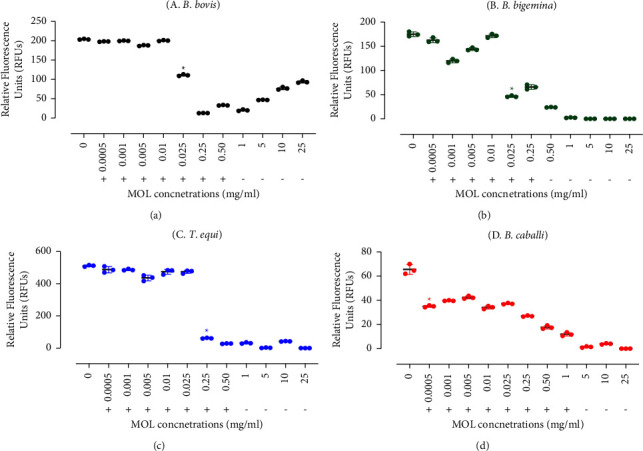
Inhibitory effect of *Moringa oleifera* leaves on *B. bovis*, *B. bigemina*, *T. equi*, and *B. caballi* on the fourth day of treatment. (a) *B. bovis*. (b) *B. bigemina*. (c) *T. equi*. (d) *B. caballi.* Each value represents the mean ± standard deviation of triplicate trials after subtraction of the background fluorescence for non-parasitized RBCs. Asterisks indicate a significant difference (*P* < 0.05) between the control- and MOL-treated cultures. MOL viability test findings for screened piroplasm parasites are indicated by +“live” and –“dead.”

MOL exhibited the greatest inhibitory effect on the growth of *B. bigemina* and *B. caballi*, followed by *B. bovis*, according to the computed IC_50_s (Table 1). 0.025 mg/mL MOL significantly inhibited (*P* < 0.05) the *in vitro* growth of *B. bovis* and *B. bigemina* (Figure 1). Furthermore, MOL treatments of 0.25 and 0.0005 mg/mL significantly inhibited (*P* < 0.05) *T. equi* and *B. caballi* growth, respectively (Figure 1). *B. bovis* and *T. equi in vitro* regrowth was inhibited at a dosage of 1 mg/mL in the subsequent viability test (Figures 1(a) and 1(c)). Notably, 0.5 mg/mL MOL inhibited *B. bigemina in vitro* regrowth (Figure 1(b)). At 5 mg/mL MOL, *B. caballi* regrowth was reduced (Figure 1(d)). The nonsignificant difference (*P* > 0.05) between the DMSO-containing positive control well and the untreated wells demonstrates that the diluent did not affect the effectiveness of the MOL methanolic extract. Furthermore, erythrocytes pretreated with a high dose of MOL methanolic extract 25 mg/mL showed no influence on erythrocyte shape as compared to non-treated erythrocytes (Figure S1).

**Figure 2:**
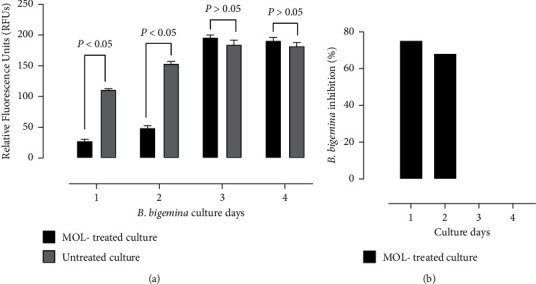
Delayed-death effect on *B. bigemina*. (a) Inhibitory effect of *Moringa oleifera* leaves (MOL) in comparison with control culture at different incubation periods after 24 h exposure to IC_99_ MOL. Each value represents the mean ± standard deviation of triplicate trials. *P* < 0.05 indicates a significant difference between the treated and the non-treated cultures. (b) Percent of inhibition in *B. bigemina* growth in comparison with non-treated culture at different incubation times after exposure to IC_99_ MOL.

To study the delayed-death effect, the test was performed on the most susceptible *Babesia* parasite to the inhibitory effect of MOL, *B. bigemina*. The parasite was treated with IC_99_ MOL for 24 hours. Then, the emitted fluorescence signals were determined. Peak fluorescence levels for untreated culture were reported at 72 and 96 hours (Figure 2(a)). At 24 hours, the IC_99_ MOL methanolic extract resulted in 75.22% growth of *B. bigemina* (Figure 2(b)). The inhibitory impact of MOL extract was then reduced to 68.16% after 48 hours.

### 3.2. DA and MMV Hits Impact the MOL Methanolic Extract's *In Vitro* Effectiveness

MOL combined with either DA or MMV was investigated against various *Babesia* species. MOL/DA combination exhibited a higher inhibitory effect on the growth of *B. bovis* and equine *Babesia/Theileria* infections versus DA monotherapy at M6 (1/4 MOL: 12 DA) and M8 (1/8 MOL: 12 DA), respectively (Table 2). Such findings confirmed MOL's potential anti-*Babesia* impact, particularly when prescribed in low dosage concurrently with DA.

MMV665941, MMV396693, MMV665810, MMV007092, or MMV666093 significantly improved the growth inhibitory effect of MOL on *B. bovis*, even at M4 (12 MOL: 1/8 MMV compounds) concentrations (Table 3). MMV006787 increased MOL's inhibitory anti-*B. bovis* activity when administered in a combined ratio of 1/2 IC_50_s of both medicines (Table 3). In the same pattern, MMV665875 improved the anti-*B. bovis* efficacy of MOL, even at M8 (1/8 of MOL leaves and MMV compound) concentration (Table 3).

### 3.3. MOL Methanolic Extract Clears *B. microti* Infection in Mice

**Figure 3:**
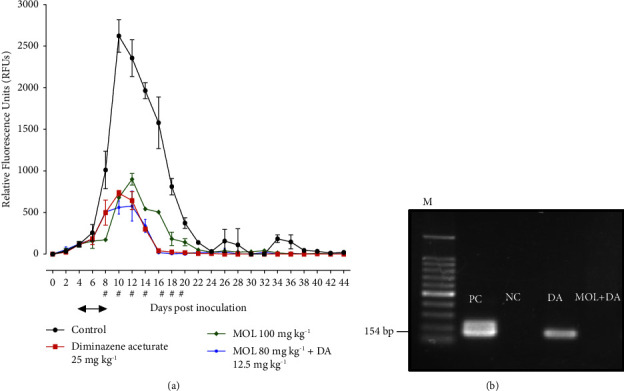
*In vivo* chemotherapeutic efficacy of *moringa oleifera* leaves (MOL), diminazene aceturate (DA), and the combination of both drugs on the growth of *Babesia microti*. (a) Drug inhibitory effects. (b) PCR of the ss-rRNA gene in the blood of *B. microti–*infected mice. All mice were intraperitoneally injected with 1 × 10^7^*B. microti*-RBCs. The treatment started when parasitemia reached approximately 1% in the infected mice and continued for 5 successive days. In the control group, mice were injected with I/P doses of DMSO in phosphate buffer saline (PBS) (0.02%). DA and MOL were administrated in subcutaneous and oral dosages, respectively. Drugs in the combination therapy were administrated during the same inoculation time. Each value represents the mean ± standard deviation of five mice per experimental group. Asterisks indicate significant differences (ANOVA; ^*∗*^*P* < 0.05) between the MOH–treated and control groups. PC, positive control; NC, negative control. *M* indicates a 100 bp DNA ladder. Treatment time is indicated by an arrow (4–8 days post inoculation).

MOL's *in vivo* inhibitory activity against *B. microti* was tested in a mouse model. When compared to the positive control group, MOL treated mice demonstrated a significant suppression (*P* < 0.05) in the generated fluorescence signals after days 8 to 20 p.i (Figure 3(a)). Peak fluorescence values in 100 mg·kg^−1^ MOL therapy and 80 mg·kg^−1^ MOL plus 12.5 mg·kg^−1^ DA were 900.43 and 578.08 at 12 days p.i, respectively (Figure 3(a)). Whereas, peak fluorescence levels in the positive control group and 25 mg·kg^−1^ DA at 10 days p.i. were 2623 and 733.14, respectively (Figure 3(a)). Treatment with 100 mg·kg^−1^ MOL monotherapy for five days resulted in 74.06% inhibition, compared to 72.04% inhibition with 25 mg·kg^−1^ DA (Figure 3(a)). At 10 and 12 days p.i, the inhibition in the fluorescence values was higher in mice treated with MOL/DA combination than in animals treated with 25 mg·kg^−1^ DA (Figure 3(a)). Oral injections of 80 mg·kg^−1^ MOL combined with a subcutaneous dosage of 12.5 mg·kg^−1^ DA inhibited the parasite growth at 10 and 12 days p.i by 78.57% and 75.47%, respectively, compared to 72.04% and 72.66% inhibitions in the presence of 25 mg·kg^−1^ DA at 10 and 12 days p.i. (Figure 3(a)).

**Figure 4:**
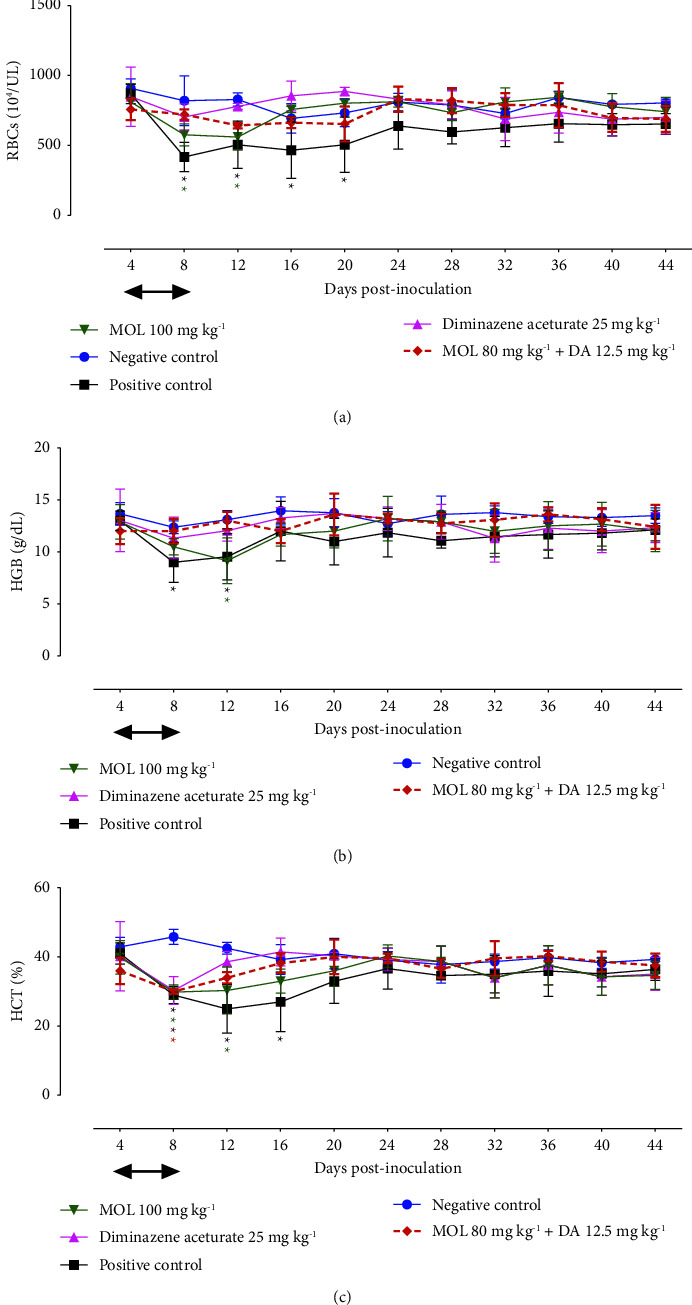
Hematological variables in *B. microti*-infected mice treated with *moringa oleifera* leaves (MOL). (a) RBCs. (b) Hemoglobin (HGB). (c) Hematocrit (HCT). Each value represents the mean ± standard deviation of five mice per experimental group. Asterisks indicate a significant difference (ANOVA; ^*∗*^*P* < 0.05) between the treated or infected mice and the uninfected mice. Treatment time is indicated by an arrow (4–8 days post inoculation).

MOL combined with a low dose of DA normalized the evaluated hematological variables almost identically to those treated with 25 mg·kg^−1^ DA (Figure 4). Such data demonstrated the antibabesial efficacy of MOL/DA combination therapy.

On day 44 post-infection, a nested PCR targeting the *B. microti ss-rRNA* gene was performed to detect parasite remains in the blood of treated mice. It should be noted that the parasite gene was not identified in the blood of mice given the MOL/DA combination therapy (Figure 3(b)). The *B. microti* residual gene, on the other hand, was found in the blood of mice administered DA alone (Figure 3(b)). These data suggest MOL/DA combo therapy's advantages over the routinely recommended antibabesial medication DA.

## 4. Discussion

In the present study, the *in vitro* and *in vivo*anti-piroplasm inhibitory effect of MOL was examined. Compared to the recently evaluated herbal antibabesial drugs, pomegranate (*Punica granatum*) peel [11], turmeric (*Curcuma longa*), and *Zingiber officinale* rhizome [9, 10], MOL exhibits lower IC_50_ values for the screened *Babesia* species (Table 1). Furthermore, the obtained data revealed that bovine or horse RBCs were unaffected by extremely high MOL methanolic extract concentrations.

In mice treated with 100 mg·kg^−1^ MOL, *B. microti* growth was reduced by 74.06%. This result is similar to 72.04% inhibition of DA (25 mg·kg^−1^), 72.4% inhibition of allicin (100 mg·kg^−1^) [17], and 77.5% inhibition of thiostrepton (500 mg·kg^−1^). Of note, MOL methanolic extract inhibits *B. microti* growth in mice better than 40.38% for *Zingiber officinale* rhizome methanolic extract [9], 68.5% for 500 mg·kg^−1^ clindamycin [16], 58.3% for 30 mg·kg^−1^ allicin [17], and 61% and 45% for 100 mg·kg^−1^ and 70 mg·kg^−1^ thymoquinone [15], and 34%, 31%, and 49% for 100 mg·kg^−1^ enoxacin, 150 mg·kg^−1^ norfloxacin, and 700 mg·kg^−1^ ofloxacin, respectively [4].

*Moringa oleifera*, a popular vegetable plant in many Asian and Southeast Asian countries, has several compounds with antioxidant, anti-cancer, anti-trypanosomal, anti-microbial, anti-inflammatory, anti-diabetic, anti-dyslipidemia, and anti-hypertensive characteristics [5, 8]. In mice infected with *P. berghei* ANKA, a watery crude extract of *M. oleifera* leaves displayed suppressive and curative antimalarial activity [18]. MOL's antibabesial activity may be explained by the near biological similarities between *Plasmodium* and *Babesia* parasites. LC-MS analysis of MOL revealed that the most abundant polyphenolic catechism found in the MOL extract was isoquercetin and rutin of chemical formulas C_21_H_20_O_12_, and C_27_H_30_O_16_, respectively (Figure S2). Other polyphenolic catechisms were detected in the extract as quercitrin, vanillina, chlorogenic acid, and quercetin (Figure S2). Notably, a previous study in our Laboratory approved the substantial antibabesial effect of quercetin [19].

MOL's *in vitro* inhibitory impact against piroplasm parasite growth was improved in our study when combined with DA. These findings are consistent with the *in vitro* inhibitory effects of myrrh oil/DA [20], allicin/DA [17], and TQ/DA combos [15]. These findings encourage us to study the inhibitory effect of MOL/DA in mice infected with *B. microti*. Interestingly, MOL/DA inhibits *B. microti* growth at a faster rate than clindamycin mixed with the natural product quinine [21]. Furthermore, MOL/DA exhibited inhibition in *B. microti* growth higher than 53.25% inhibition rates for 85 mg·kg^−1^ PYR combined with 10 mg·kg^−1^ DA [9], 67% inhibition rates for 50 mg·kg^−1^ enoxacin and 10 mg·kg^−1^ DA [4], and 62.5% inhibition rates for 50 mg·kg^−1^ oral dosage of TQ and 10 mg·kg^−1^ subcutaneous dose of DA [15].

1. Nested PCR experiment was used to test the ability of MOL/DA combined therapy to further remove parasite nucleic acid from the animal's blood. Combination therapy was effective in removing parasite nucleic acid from the blood of treated mice. Such finding is similar with the efficacy of enoxacin/DA [4], PYR/DA [9].
2. *Bovis in vitro* growth suppression was increased after treatment with MOL/MMV compounds, particularly MOL/MMV665875. Rizk et al. [13] revealed that the cysteine protease (CP) gene could be one of the targets of MMV665875 (Probe similar chemical) for *in vitro* growth inhibition of several protozoan parasites. CPs are ubiquitous in all living species and are required for many protozoan parasites to enter the cells of their hosts [11, 22]. For *Plasmodium*, CPs play an important role in parasite egression by degrading both hemoglobin and erythrocyte cytoskeletal proteins, causing the infected erythrocyte to rupture [10]. As a result, CPs provide interesting new therapeutic targets for a variety of protozoan illnesses, including trypanosomiasis [10], malaria [11], schistosomiasis [23], and leishmaniasis [24]. A prior in vitro investigation [20] for piroplasm parasites showed the presence of CPs in *B. bovis.* Following that, the genome project [25] established the existence of CPs in *Babesia* and described CP in parasites of *B. bovis.* Furthermore, Okubo et al. [20] proposed that CPs play an important role in *B. bovis* invasion of host RBCs. Furthermore, Ascencio et al. [26] revealed that *T. equi* had an unusually large number of C1A-CP paralogs, which could be associated with the evolution of the schizont stage. However, the precise role of babesial CPs is unknown. The findings suggest that MOL may be targeted to the CP gene in the *Babesia* parasite. Further investigation is required to determine the mechanism of improvement in the inhibitory impact of MOL when combined with the screened MMV hits, particularly MMV665875. Although the current study demonstrated an improvement in the *in vitro* inhibitory effect of MOL when combined with different MMV hits from the malaria box on *B. bovis* growth, the synergetic or antagonistic relationships between these combination therapies against the growth of *B. microti* in mice have not yet been evaluated. As a result, additional research is required to assess the potential inhibitory effects of these combinations on the growth of *B. microti* in a mouse model.

## 5. Conclusions

*Babesia bigemina* and *B. caballi* were the most sensitive parasites to the *in vitro* inhibitory effect of MOL, followed by *B. bovis*. MOL in combination with MMV665875 exhibited the highest inhibitory efficacy against *B. bovis* growth. *In vitro*, the combination of MOL and DA suppressed piroplasm growth, particularly in equine piroplasm parasites. Emitted fluorescence signals were significantly reduced in mice treated with a combination treatment consists of lower doses of MOL and DA. Furthermore, the MOL/DA combination therapy was effective in eliminating the *B. microti *ss-rRNA gene from treated mice's blood. The obtained findings revealed that MOL may be effective in the treatment of animal piroplasmosis, particularly when combined with a low dose of either MMV665875 or DA.

## Figures and Tables

**Table 1:** IC_50_ values of *Moringa oleifera* leaves evaluated for bovine *Babesia* and equine *Babesia* and *Theileria* parasites.

Organism	IC_50_ (*µ*g/ml)^a^				
	MOL	DA	*Zingiber officinale* rhizome^b^	Turmeric (*Curcuma longa*)^c^	Pomegranate (*Punica granatum*) peel^d^
*B. bovis*	45.29 ± 6.14	0.16 ± 0.02	588 ± 23.80	830 ± 78	154.45 ± 23.11
*B. bigemina*	19.16 ± 0.45	0.08 ± 0.003	14800 ± 1240	ND	40.90 ± 9.35
*T. equi*	137.49 ± 16.07	0.28 ± 0.01	39350 ± 1340	1405 ± 575	100 ± 16.20
*B. caballi*	9.29 ± 0.014	0.012 ± 0.003	356.05 ± 34.71	720 ± 90	77.27 ± 16.94

^a^The final IC_50_s obtained represents the mean ± SD of values obtained from three separate experiments. ^b^[9], ^c^[10], ^d^[11]. MOL, *Moringa oleifera* leaves methanolic extract. DA, diminazene aceturate. ND not detected.

**Table 2:** Growth inhibitory efficacy of *Moringa oleifera* leaves/diminazene aceturate combinations on bovine *Babesia* and equine *Babesia* and *Theileria* infections.

Group	Fluorescence values (mean ± SD)			
	*B. bovis*	*B. bigemina*	*T. equi*	*B. caballi*
Control	253.07 ± 5.09	223.62 ± 1.37	311.95 ± 9.79	296.04 ± 1.88
DA IC_50_	115.50 ± 0.17	117.92 ± 2.66	149.45 ± 0.21	126.63 ± 0.49
*M*1 (3/4 : 3/4)	16.78 ± 0.63^*∗∗*^	115.90 ± 5.57^*∗*^	1.04 ± 1.14^*∗∗*^	71.02 ± 0.57^*∗∗*^
*M*2 (3/4 : 1/2)	27.91 ± 0.09^*∗∗*^	150.28 ± 3.10^*∗*^	7.64 ± 1.15^*∗∗*^	86.92 ± 0.31^*∗∗*^
*M*3 (1/2 : 3/4)	27.32 ± 0.28^*∗∗*^	159.27 ± 1.53^*∗*^	13.64 ± 0.83^*∗∗*^	89.12 ± 0.29^*∗∗*^
*M*4 (1/2 : 1/2)	27.97 ± 0.44^*∗∗*^	160.87 ± 2.38^*∗*^	15.48 ± 0.83^*∗∗*^	89.85 ± 1.10^*∗∗*^
*M*5 (1/4 : ¾)	70.56 ± 0.41^*∗∗*^	171.57 ± 1.72^*∗*^	19.14 ± 0.58^*∗∗*^	91.79 ± 0.69^*∗∗*^
*M*6 (1/4 : 1/2)	95.48 ± 1.85^*∗∗*^	194.73 ± 2.54^*∗*^	21.36 ± 0.83^*∗∗*^	97.85 ± 1.31^*∗∗*^
*M*7 (1/8 : 3/4)	142.13 ± 1.37^*∗*^	199.22 ± 0.55^*∗*^	36.29 ± 1.36^*∗∗*^	114.99 ± 1.70^*∗∗*^
*M*8 (1/8 : 1/2)	150.31 ± 1.62^*∗*^	202.10 ± 1.06^*∗*^	36.19 ± 0.24^*∗∗*^	115.05 ± 3.45^*∗∗*^

^*∗*^ *P* < 0.05 statistically significant differences between the mixed-drug-treated and control groups only. ^*∗∗*^*P* < 0.05 statistically significant differences between the mixed-drug-treated group and both the diminazen aceturate and control groups. *M*1–8 refer to the combinations of *Moringa oleifera* leaves (MOL): DA, diminazen aceturate.

**Table 3:** Growth inhibitory efficacy of *Moringa oleifera* leaves and highly potent MMV compounds combinations on *B. bovis* infection.

Group	Fluorescence values (mean ± SD)							
MMV compounds	MMV665941	MMV396693	MMV006787	MMV665810	MMV007092	MMV085203	MMV666093	MMV665875
Control	260.31 ± 2.68							
MMV IC_50_	114.18 ± 2.42	124.09 ± 1.44	133.49 ± 4.16	117.51 ± 3.01	129.22 ± 3.06	115.27 ± 2.18	122.88 ± 4.57	128.03 ± 5.77
*M*1 (3/4 : 1/2)	13.31 ± 0.24^*∗∗*^	18.02 ± 0.71^*∗∗*^	12.07 ± 1.62^*∗∗*^	14.70 ± 1.23^*∗∗*^	22.56 ± 0.39^*∗∗*^	23.95 ± 0.32^*∗∗*^	9.09 ± 0.36^*∗∗*^	11.54 ± 1.05^*∗∗*^
*M*2 (3/4 : 1/8)	30.21 ± 0.27^*∗∗*^	27.99 ± 0.58^*∗∗*^	25.43 ± 2.48^*∗∗*^	20.19 ± 3.22^*∗∗*^	34.12 ± 1.47^*∗∗*^	35.99 ± 0.82^*∗∗*^	21.77 ± 4.51^*∗∗*^	13.85 ± 0.81^*∗∗*^
*M*3 (1/2 : 1/2)	48.12 ± 6.63^*∗∗*^	114.05 ± 2.43^*∗∗*^	91.00 ± 0.25^*∗∗*^	96.90 ± 6.26^*∗∗*^	72.25 ± 0.48^*∗∗*^	121.17 ± 1.51^*∗*^	84.73 ± 0.64^*∗∗*^	23.80 ± 0.65^*∗∗*^
*M*4 (1/2 : 1/8)	109.23 ± 1.57^*∗∗*^	117.59 ± 0.42^*∗∗*^	156.23 ± 6.36^*∗∗*^	100.55 ± 0.32^*∗∗*^	116.81 ± 2.59^*∗∗*^	133.47 ± 0.28^*∗*^	102.63 ± 0.07^*∗∗*^	25.98 ± 1.09^*∗∗*^
*M*5 (1/4 : 1/2)	113.83 ± 2.11^*∗*^	165.22 ± 0.10^*∗*^	158.00 ± 2.79^*∗*^	121.70 ± 0.32^*∗*^	123.69 ± 2.14^*∗*^	172.62 ± 1.84^*∗*^	152.17 ± 0.21^*∗*^	40.92 ± 0.54^*∗∗*^
*M*6 (1/4 : 1/8)	127.75 ± 2.50^*∗*^	176.15 ± 1.04^*∗*^	168.15 ± 3.01^*∗*^	124.13 ± 0.28^*∗*^	138.29 ± 0.40^*∗*^	201.90 ± 0.78^*∗*^	163.78 ± 1.34^*∗*^	66.44 ± 0.77^*∗∗*^
*M*7 (1/8 : 1/2)	130.85 ± 0.92^*∗*^	177.29 ± 0.67^*∗*^	184.13 ± 0.42^*∗*^	170.58 ± 0.71^*∗*^	137.40 ± 0.46^*∗*^	203.00 ± 3.98^*∗*^	161.35 ± 0.04^*∗*^	83.30 ± 1.11^*∗∗*^
*M*8 (1/8 : 1/8)	163.58 ± 2.9^*∗*^	177.77 ± 3.52^*∗*^	187.85 ± 1.59^*∗*^	175.28 ± 0.21^*∗*^	158.70 ± 0.71^*∗*^	210.12 ± 1.31^*∗*^	187.58 ± 1.06^*∗*^	102.92 ± 1.17^*∗∗*^

^*∗*^ *P* < 0.05 statistically significant differences between the mixed-drug-treated group and the control group. ^*∗∗*^*P* < 0.05 statistically significant differences between the mixed-drug-treated group and both MMV hits-treated groups and the control group. *M*1–8 refer to the mixtures of MOL: MMV compounds.

## Data Availability

Necessary data are available in the manuscript. Additional information can be received from the corresponding authors upon reasonable request.

## Supplementary Materials

Supplementary Materials Supplementary 1. Figure S1. Microscopy picture for bovine erythrocytes treated with MOL methanolic extract 25 mg/mL. Supplementary 2. Figure S2. Liquid Chromatography Mass Spectrometry analysis of *Moringa oleifera* Leaves. At 0.3 mg/mL, standard stock solutions were created in methanol. Individual standard stock solutions were diluted with methanol to produce working standard solutions. A controller, two pumps, a degasser, and an autosampler were included in the Acquity I-class UPLC system (Waters Corp., Milford, MA, USA) used for liquid chromatography. On an ACQUITY UPLC BEH C18 column (100 mm 2.1 mm, 1.7 m) kept at 30 C, polyphenol separation was completed. The flow rate was 0.3 mL/min, and the injection volume was 1 L. The autosampler was set at 10 C. In negative ionization mode (ESI), a Xevo tandem quadruple detector (TQD) mass spectrometer (Waters Corp., Milford, MA, USA) was used. Supplementary 3. Figure S3. Chemical structures of the most abundant polyphenolic catechism in MOL extract identified by Liquid Chromatography Mass Spectrometry analysis.
