# Supplementary material for: Evaluation of the Inhibitory Effect of Moringa oleifera Leaves Methanolic Extract against In Vitro Growth of Several Babesia Species and Theileria equi and the In Vivo Growth of Babesia microti
Source: J Trop Med. 2023 Oct 31;2023:4285042. doi: 10.1155/2023/4285042 (PMC10630014; doi:10.1155/2023/4285042)
Supplement: Supplementary Materials — Supplementary 1. Figure S1. Microscopy picture for bovine erythrocytes treated with MOL methanolic extract 25 mg/mL. Supplementary 2. Figure S2. Liquid Chromatography Mass Spectrometry analysis of Moringa oleifera Leaves. At 0.3 mg/mL, standard stock solutions were created in methanol. Individual standard stock solutions were diluted with methanol to produce working standard solutions. A controller, two pumps, a degasser, and an autosampler were included in the Acquity I-class UPLC system (Waters Corp., Milford, MA, USA) used for liquid chromatography. On an ACQUITY UPLC BEH C18 column (100 mm 2.1 mm, 1.7 m) kept at 30 C, polyphenol separation was completed. The flow rate was 0.3 mL/min, and the injection volume was 1 L. The autosampler was set at 10 C. In negative ionization mode (ESI), a Xevo tandem quadruple detector (TQD) mass spectrometer (Waters Corp., Milford, MA, USA) was used. Supplementary 3. Figure S3. Chemical structures of the most abundant polyphenolic catechism in MOL extract identified by Liquid Chromatography Mass Spectrometry analysis. [file 4285042.f1.docx]

**Supplementary data**

Fig. S1. Microscopy picture for bovine erythrocytes treated with MOL methanolic extract 25 mg/mL


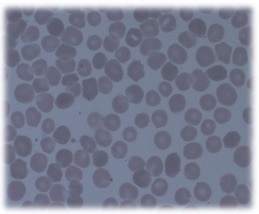

**Fig. S2.** Liquid Chromatography Mass Spectrometry analysis of *Moringa oleifera* Leaves. At 0.3 mg/mL, standard stock solutions were created in methanol. Individual standard stock solutions were diluted with methanol to produce working standard solutions. A controller, two pumps, a degasser, and an autosampler were included in the Acquity I-class UPLC system (Waters Corp., Milford, MA, USA) used for liquid chromatography. On an ACQUITY UPLC BEH C18 column (100 mm 2.1 mm, 1.7 m) kept at 30 C, polyphenol separation was completed. The flow rate was 0.3 mL/min, and the injection volume was 1 L. The autosampler was set at 10 C. In negative ionization mode (ESI), a Xevo tandem quadruple detector (TQD) mass spectrometer (Waters Corp., Milford, MA, USA) was used.

**Fig. S3.** Chemical structures of the most abundant polyphenolic catechism in MOL extract identified by Liquid Chromatography Mass Spectrometry analysis
